# Local public health workers' perceptions toward responding to an influenza pandemic

**DOI:** 10.1186/1471-2458-6-99

**Published:** 2006-04-18

**Authors:** Ran D Balicer, Saad B Omer, Daniel J Barnett, George S Everly

**Affiliations:** 1 Epidemiology Department, Faculty of Health Sciences, Ben-Gurion University of the Negev, Be'er Sheva, Israel; 2 Department of International Health, Johns Hopkins Bloomberg School of Public Health, Baltimore, Maryland, USA; 3 Johns Hopkins Center for Public Health Preparedness, Department of Environmental Health Sciences, Johns Hopkins Bloomberg School of Public Health, Baltimore, Maryland, USA

## Abstract

**Background:**

Current national preparedness plans require local health departments to play an integral role in responding to an influenza pandemic, a major public health threat that the World Health Organization has described as "inevitable and possibly imminent". To understand local public health workers' perceptions toward pandemic influenza response, we surveyed 308 employees at three health departments in Maryland from March – July 2005, on factors that may influence their ability and willingness to report to duty in such an event.

**Results:**

The data suggest that nearly half of the local health department workers are likely not to report to duty during a pandemic. The stated likelihood of reporting to duty was significantly greater for clinical (Multivariate OR: 2.5; CI 1.3–4.7) than technical and support staff, and perception of the importance of one's role in the agency's overall response was the single most influential factor associated with willingness to report (Multivariate OR: 9.5; CI 4.6–19.9).

**Conclusion:**

The perceived risk among public health workers was shown to be associated with several factors peripheral to the actual hazard of this event. These risk perception modifiers and the knowledge gaps identified serve as barriers to pandemic influenza response and must be specifically addressed to enable effective local public health response to this significant threat.

## Background

Local health departments are considered the backbone of public health response plans for any and all infectious disease outbreaks. An influenza pandemic is considered increasingly likely, and is now considered one of the most significant and urgent threats to the nation's public health preparedness infrastructure. It has been argued that of the 12 disaster scenarios recently assessed by the U.S. Department of Homeland Security, pandemic influenza is the most likely and perhaps the most deadly[1]. The United States pandemic influenza plan released in November 2005, lays out a critical role for local and state public health agencies during a pandemic, including: providing regular situational updates for providers; providing guidance on infection control measures for healthcare and non-healthcare settings; conducting or facilitating testing and investigation of pandemic influenza cases; and investigating and reporting special pandemic situations[2].

These specified activities would require an extensive prompt response by local health departments. Current contingency plans account for possible personnel shortages due to influenza morbidity, but previous studies have shown that during extreme scenarios, a varying proportion of healthcare workers may be unable or unwilling to report to duty [3-5]. This may be even truer for health departments, where unlike more "traditional" first responder agencies (such as law enforcement, fire services, and emergency medical services), the capacity and willingness to respond 24/7 to crises is not historically ingrained in the workforces' professional cultures and training. Even in the post-9/11 environment, recent data indicate inconsistent and sometimes slow after-hours response by health departments to urgent events involving communicable disease[6].

Risk perception theory provides a revealing framework for better understanding response limitations and needs of the public health workforce. The perceived risk, according to this theory, is a multifactorial phenomenon, involving the summation of actual risk and other peripheral influences independent of the actual risk, such as perceived authority, trust, and situational control; these peripheral influences have been termed "outrage"[7] or "dread."[8].

Based on these models, it was previously suggested that contributing factors peripheral to the actual risk will have a considerable practical impact on how public health employees would respond in a crisis[9]. Aside from physical and circumstantial barriers such as availability of transportation or dependency of family members, we have identified specific risk perception issues whose impact may be markedly high and of unique importance for the public health workforce's response to a crisis. These factors, or modifiers, stem from a number of features previously suggested to have been associated with elevated risk perception, including manageability of the threat; risk to future generations; direct personal impact; and sense of control over events.

Based on these modifiers, several major barriers to effective public health workforce emergency response were suggested; these include uncertainty regarding working environment safety, unclear expectations of role-specific emergency response requirements, safety and well being of family members, inadequate emphasis on the critical value of each employee to the agency response efforts, and insufficient emphasis on stress management techniques – all of which may heighten employees' sense of dread due to a lack of personal control[9].

In light of the projected impact of an influenza pandemic, health departments must optimize the response rate of their employees in this crisis scenario. Based on the emergency response principle that all disasters are "local"[10], we have set out to assess local public health employees' risk perception and likelihood of reporting to duty during a local outbreak of pandemic influenza, and to uncover the variables that affect these outcomes, thus providing a needed evidence base for health departments' planning and training efforts.

## Methods

We conducted the study in Carroll, Dorchester, and Harford county health departments between March 2005 and July 2005. All three health departments are located in Maryland, and range in size from 132 employees to 225 employees. We selected these health departments because of their location in communities ranging from 30,000 on Maryland's Eastern Shore (Dorchester County) to 235,000 in the greater Baltimore/Towson metropolitan area (Harford County)[11], thus reflecting the 96% of the nation's local public health agencies serving communities with populations of 500,000 or fewer[12]. Self- administered anonymous surveys were sent to all health department personnel by their respective health departments. Completed surveys were directly mailed to investigators at the Johns Hopkins Center for Public Health Preparedness.

### Survey content

The survey included questions on personal characteristics such as job classification, gender, and age. The respondents used a 5-point Likert scale for questions pertaining to a possible flu pandemic: probability of them reporting to work ("very likely" to "not at all likely"); possibility of being asked by their health department to respond to an emergency ("very likely" to "not at all likely"); how knowledgeable they thought they were about the potential public health impact of pandemic influenza ("very knowledgeable" to "not at all knowledgeable"); how confident they were about being safe in their work roles ("very confident" to "not at all confident"); how likely was their family prepared to function in their absence ("very likely" to "not at all likely"); how likely they felt their health department would provide them with timely updates ("very likely" to "not at all likely"); how familiar they were with their role specific response requirements ("very familiar" to "not at all familiar"); how well they thought they could address the questions of a concerned member of the public ("very well" to "not at all"); how significant a role they thought they would play in the agency's overall response ("very significant" to "not at all significant"); how important would be pre-event preparation and training ("very important" to "not at all important"); how important it was for them to have psychological support available during the event ("very important" to "not at all important"); and how important it was for them to have psychological support available after the event ("very important" to "not at all important").

The job classification variable was collapsed into technical/support staff (such as computer entry staff, clerical staff (e.g. receptionists), computer specialists, health information systems data analysts etc.), and professional staff. The latter included public health officials, clinical staff (e.g., nurse, dentist, physician), public health communicable disease staff, environmental health staff, public information staff, and other public health professional staff (e.g., health educator, legal professional, financial officer, other).

### Data analysis

We dichotomized the responses to the job classification question into professional and technical/support categories. Questions about likelihood of reporting to work and pandemic influenza-related attitudes and beliefs were dichotomized into responses with a score two or less, and all other responses. We used logistic regression to compute Odds Ratios to evaluate the association of demographic variables and attitudes and beliefs with self-described likelihood of reporting to work. We used multivariate logistic regression to explore associations between attitudes and beliefs related to pandemic influenza preparedness and self-described likelihood of reporting to work. The model included adjustment for age, gender, and job classification. Similarly, we used bivariate and multivariate (adjusted for age, gender, and job classification) logistic regression models to evaluate the association between the various attitudes and beliefs. In order to assess non-response bias, we compared age, gender, and job classification distributions for the respondents and for all health department personnel.

We used TeleForm Version 8 (Cardiff, Vista, CA) and Stata Version 9 (Stata Corporation, College Station, TX) for data capturing and analysis respectively.

## Results

We received 118 out of 205 (57.6%), 74 out of 128 (57.8%), and 116 out of 198 (58.6%) surveys fromCarroll, Dorchester, and Harford county health departments respectively, resulting in an overall response rate of 58.0% (n = 308). We did not find a statistically significant difference in age and gender distribution between the respondents and all health department personnel. A small yet statistically significant difference in the proportion of technical/support staff (vs. professional staff) was detected (22.4% vs. 32% in the study group and all personnel respectively, p = 0.003), yet no significant difference in the proportions of professional staff subgroups was detected.

Of the 303 who responded to the question about their likelihood of reporting during a pandemic influenza related emergency, 163 (53.8%) indicated they would likely report to work during such an emergency. Age and gender did not have an association with likelihood of reporting. Clinical staff indicated a higher likelihood of reporting (Multivariate OR: 2.5; CI 1.3–4.7) than technical/support staff (Table 1).

**Figure 1:**
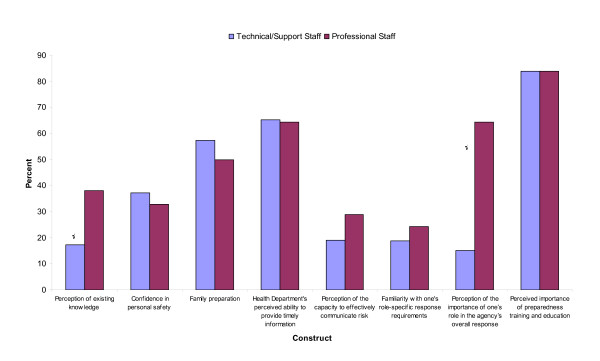
Proportion of individuals who agreed with each of the attitude and belief constructs by staff type.

Only 40% of all respondents- 45.1% professional staff and 26.1% technical/support staff – felt it was likely they would be asked by their health department to respond to a pandemic influenza related emergency. Perception of likely to be asked by the Health Department to respond was associated with self-described likelihood of reporting (Multivariate OR : 8.5; 95%CI 4.6–15.6). Only 33.4% (101) individuals thought of themselves to be knowledgeable about the public health impact of pandemic influenza (Table 2). Perception of one's existing knowledge about pandemic influenza, and perception of having an important role in the agency's overall response were significantly higher among professional staff compared to technical/support staff (Figure 1),

In multivariate analysis, increased self-described likelihood of reporting to work during an influenza pandemic emergency was significantly associated with agreement with several constructs, most notably perception of the capacity to communicate risk effectively, perception of the importance of one's role in the agency's overall response, and familiarity with one's role-specific response requirements in a pandemic influenza related emergency. (Table 2).

The vast majority (83%) of the respondents felt they would benefit from additional training activities. A lower perceived level of familiarity with one's role was not significantly associated with a higher perceived need for additional training (Multivariate OR: 1.4; CI 0.6–3.4). Most of the respondents also perceive psychological support during the event (57.1%) and post-event psychological support (61.3%) as important. Psychological support during and after the event was deemed more important by staff who considered themselves likely to be asked to report to duty during an event (Multivariate OR [CI]: 2.4 [1.4–4.2] and 2.8 [1.6–4.8], respectively).

Sixty-six percent of the respondents perceived themselves to be at personal risk when performing their duties during such an event. Confidence in personal safety was associated with several constructs independently of one's job classification, including perception of existing knowledge about public health impact of pandemic influenza (Multivariate OR: 4.1; CI 2.3–7.6); family preparation (Multivariate OR: 2.5; CI 1.4–4.3); health department's perceived ability to provide timely information (Multivariate OR: 5.4; CI 2.7–10.7); perception of the capacity to effectively communicate risk (Multivariate OR: 4.8; CI 2.6–9.0); perception of the importance of one's role in the agency's overall response (Multivariate OR: 4.1; CI 2.9–7.7); and familiarity with one's role-specific response requirements (Multivariate OR: 3.5; CI 1.8–6.2).

**Figure 2:**
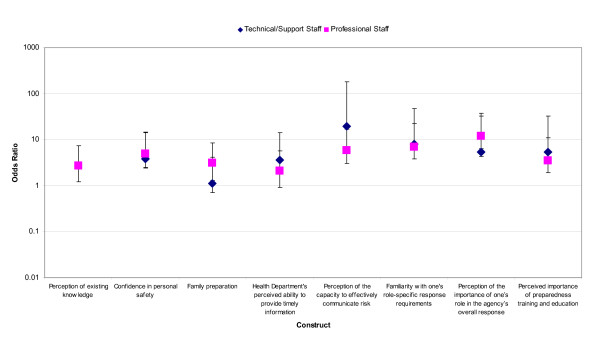
Odds Ratios of reporting to work in case of a pandemic-influenza-related emergency by staff and attitude or belief construct.

The associations between self-identified likelihood of reporting to work and perception of one's capacity to effectively communicate risk were substantially stronger for technical/support staff compared to professional staff (Bivariate OR [CI]: 19.4 [2.4–160.4] vs. 5.9 [2.9–12.2], respectively) (Figure 2).

## Discussion

The World Health Organization has urged all countries to prepare for the next influenza pandemic, which it termed in mid-2004 "inevitable and possibly imminent"[13]. The federally adopted U.S. model of all-hazards emergency readiness has presented local health departments with new organizational challenges and learning curves. The all-hazards approach entails an ability and willingness to respond to a broad spectrum of disasters, ranging from the intentional (e.g., chemical, biological, or radiological terror) to the naturally occurring (e.g., weather-related crises or non-bioterrorism related infectious disease)[14].

Current national contingency plans account for possible personnel shortages within the healthcare and public health settings, mainly due to the expected influenza morbidity among workers. Yet our data suggest that regardless of the expected morbidity among personnel during an influenza pandemic, nearly half of the local health department workers are likely not to report to duty during such an extreme public health crisis. In fact, most of the workers (and nearly three out of four technical/support workers) do not believe they will even be asked to report to work.

We have found that the willingness to report to duty during a pandemic varies considerably according to the individual's job classification. Clinical staff state they are significantly more likely to report to duty, compared with all other workers. This difference correlates well with the single most influential construct associated with willingness to report to duty – the perception of the importance of one's role in the agency's overall response. Less than a third of the respondents believed they will have an important role in the agency's response to local outbreaks of pandemic influenza, but within this subgroup, willingness to report to duty was as high as 86.8%. Belief in the importance of one's role was lowest among technical/support staff, environmental health staff, and other non-clinical professional staff (15.1%, 18.4% and 18.8% respectively), groups in which willingness to report was shown to be lowest. We therefore believe further efforts must be directed at ensuring that all local public health workers, but most notably non-clinical professional staff, understand in advance the importance of their role during an influenza pandemic – otherwise they will fail to show up when they are most needed.

Our findings fit well in the theoretical framework emphasizing risk communication needs of public health workers, who themselves serve as risk communicators[9]. Several factors, previously suggested to be risk perception "modifiers" [9] of substantial impact on public health workforce's response to a crisis, indeed proved to be important in this context. Lack of knowledge, ambiguity regarding one's exact tasks, and questionable ability in performing one's role as risk communicator were all significantly associated with a higher perceived personal risk and a two- to ten-fold decrease in willingness to report to duty; these factors proved to be more influential even than the perceived level of family preparedness to function in one's absence. It is therefore important to recognize that public health employees, who are intended to serve as purveyors of risk communication for their communities, themselves represent a community with specific perceptions that must be addressed in the context of emergency readiness training.

The threat of an impending influenza pandemic is not a new one – pandemics have been taking place once every several decades for over 300 years. Yet it was only in the last couple of years, as highly pathogenic H5N1 strain became increasingly endemic in southeast Asia and as lethal infections with the virus occurred in an alarmingly increasing rate among humans, that the urgency of the situation was openly declared by national and international health authorities. The rapidity of this evolving situation may serve to explain why only one third of the respondents felt they were adequately knowledgeable on pandemic influenza, and why only one in five respondents felt capable in effectively communicating pandemic risks. This finding is especially noteworthy, in that members of the public health support staff may become frontline telephone risk communicators in a crisis, serving as the first points of interface for concerned callers contacting a health department. Only one of the 35 technical/support staff workers who felt incapable of effective risk communication was willing to report to duty, even though most of them believed the health department will have the ability to provide timely information.

The study has some relevant limitations that must be factored into the overall analysis. First, the sample was limited to three non-randomly selected health departments, none of which serves a community larger than 250,000 residents, and all of which have staff sizes under 250. The sample size of 308 survey employees limited this study's power. As the study includes Maryland health departments only, it does not account for potential jurisdictional or regional variations nationwide in response capacity or risk perceptions toward pandemic influenza response. Furthermore, the job classifications – based on those used to develop the CDC-adopted emergency preparedness competencies[15] – do not necessarily map neatly onto functional responsibilities in disaster response. For example, health educators may play as frontline a role as clinical staff, in terms of their degree of interface with the public in a disaster. Our job categories therefore do not necessarily reflect the relative impacts of job-specific cohorts on disaster response in the event that they do not report to work.

We assessed the presence and the direction of non-response bias by comparing the distribution of personal characteristics for the respondents and for all health department personnel. The lack of significant difference in age and gender distribution, as well as the lack of significant difference in job classification other than technical/support staff indicates that the extent of such a bias in the study is probably limited. The small yet statistically significant over-representation of technical/support staff in our study group may potentially have caused a slight underestimation of overall willingness to report. However, as the internal associations between the various variables were also studied separately for the technical/support staff and professional staff (Figure 2), this over-representation should not impact the general conclusions presented above.

Having accounted for these limitations, it is important to note that the findings were internally consistent among the three surveyed health departments. Although none of the health departments served large metropolitan areas and all had fewer than 250 employees, it must also be recognized that only 4% of the nation's local health departments serve populations of 500,000 or more, and that local public health agencies tend to have small staff sizes (with a median of 13 full time employees)[12].

Interestingly, our findings show similar patterns to data on the willingness of urban healthcare workers from non-public health settings to respond to emergencies: a survey of 6248 employees from 47 healthcare facilities in the New York City area revealed that these workers were least willing (48%) to report to duty during an untreatable naturally-occurring infectious disease outbreak affecting their facility (SARS), compared to other disaster scenarios[5]. In our study we have detected similar rates of likelihood to report to duty, although lower rates could have been expected in our study population since the New York City survey focused on healthcare workers whose organizational cultures are historically much more accustomed than that of local public health workers to emergency response, in a city with a heightened awareness of disaster preparedness in the wake of the World Trade Center attacks and subsequent anthrax attacks[5].

In the face of a pandemic influenza threat, local health department employees' unwillingness to report to duty may pose a threat to the nation's emergency response infrastructure. Addressing the specific factors associated with this unwillingness is necessary to help ensure that existing local health department preparedness competencies[15] will translate into the scope of response described in the nation's pandemic influenza plans[2]. Interventions suggested to enhance the willingness of healthcare workers in non-public health department settings to report to duty in disasters include workforce preparedness education[5], provision of appropriate personal protective equipment, [4,14] crisis counseling, family preparedness and social support[5,16].

These recommendations fit well within the framework of our findings, and we further recommend that such education programs include specialized training emphasizing the specific nature of, and guidelines for, one's role in response to pandemic influenza; the relevance of each worker's role in the effectiveness of an overall public health response; and the workers' ability to provide effective risk communication. Additional research must further focus on best practice models for addressing the above described gaps in local public health response to this urgent public health threat.

## Conclusion

These data offer a current, evidence-based window into the needs of public health workers who would serve as a backbone of locally-driven emergency response in an influenza pandemic setting. We found that most of these workers feel they will work under significant personal risk, in a scenario they are not adequately knowledgeable about, performing a role they are not sufficiently trained for, and believing this role does not have a significant impact on the agency's overall response. These specific perceptions and needs must be attended, and specific intervention programs must be initiated. In order to reduce the perceived risk associated with the worker's role in an influenza pandemic, each worker must have better understanding of the scenario and importance of his or her personal role within these settings, confidence that the agency will provide adequate protective equipment for its employees, psychological support and timely information, and a belief of being well-trained to cope with emergency responsibilities including the ability to communicate risk to others. In view of what is currently considered to be an impending influenza pandemic, a wide gap between these desired targets and current status exists, that may lead to significant hindrance in the ability of local health departments to function adequately.

## Competing interests

The author(s) declare that they have no competing interests.

## Authors' contributions

RDB was the lead writer and coordinator on the manuscript, and worked on the content development and distribution of the survey instrument. SBO coordinated data analysis and wrote the Methods and Results sections. DJB contributed to writing survey instrument content, coauthored the Background, Discussion, and Conclusions sections, and co-edited the manuscript. GSE Jr. developed the survey instrument content, coedited the manuscript, and provided guidance on the development of the Discussion and Conclusions sections.

## Pre-publication history

The pre-publication history for this paper can be accessed here:



## Figures and Tables

**Table 1:** Demographic characteristics of the study population

		**Likelihood of Reporting**	
**Characteristic**	**n(%)**	**Bivariate OR (95%CI)**	**Multivariate OR†(95%CI)**
**Age**			
20–30	20 (6.6)	Reference	Reference
30–40	48 (15.8)	1.2 (0.4–3.4)	0.9 (0.3–2.8)
40–50	102 (33.6)	1.3 (0.5–3.3)	0.8 (0.3–2.5)
50–60	107 (35.2)	1.3 (0.5–3.3)	0.9 (0.3–2.5)
Over 60	27 (8.9)	0.9 (0.3–3)	0.5 (0.1–1.9)
**Gender**			
Male	51 (17)	Reference	Reference
Female	249 (83)	0.7 (0.4–1.4)	0.6 (0.3–1.2)
**Job Classification**			
Technical/Support Staff	69 (22.4)	Reference	Reference
Public health official	7 (2.3)	2.6 (0.5–14.2)	1.9 (0.3–11)
Clinical staff (e.g., nurse, dentist, physician)	102 (33.1)	2.3 (1.2–4.4)	2.5 (1.3–4.7)
Public health communicable disease staff	12 (3.9)	3.1 (0.8–12.4)	3 (0.7–12.1)
Environmental health staff	39 (12.7)	0.9 (0.4–1.9)	0.6 (0.2–1.4)
Public Information Staff	8 (2.6)	0.3 (0.1–1.8)	0.4 (0.1–1.9)
Other Public Health Professional Staff (e.g., health educator, legal professional, financial officer, other)	71 (23.1)	0.7 (0.3–1.3)	0.6 (0.3–1.1)

†Adjusted for Age, Gender, Job Classification

**Table 2:** Associations of attitudes and beliefs regarding pandemic influenza preparedness with projected likelihood of reporting to duty by local health department personnel.

	**Construct Agreement n(%)**	**Bivariate OR (95%CI)**	**Multivariate Model†OR (95%CI)**
Perception of existing knowledge about public health impact of pandemic influenza	101 (33.4)	3.5 (2.1–5.9)	3.1 (1.8–5.5)
Confidence in personal safety	100 (33.8)	4.4 (2.6–7.6)	4 (2.2–7.2)
Family preparation	155 (51.7)	2.4 (1.5–3.8)	2.1 (1.2–3.4)
Health Department's perceived ability to provide timely information	195 (64.6)	2.4 (1.5–3.8)	2.3 (1.3–3.8)
Perception of the capacity to effectively communicate risk	80 (26.6)	7.1 (3.6–13.9)	6.6 (3.2–13.5)
Familiarity with one's role-specific response requirements	71 (23.1)	7.2 (3.5–14.7)	7.6 (3.4–16.9)
Perception of the importance of one's role in the agency's overall response	93 (31.1)	10.4 (5.3–20.3)	9.5 (4.6–19.9)
Perceived importance of preparedness training and education	254 (83.8)	3.8 (1.9–7.5)	3.4 (1.6–7.1)

* A score of 4 or 5 on the likert-type scale

† Adjusted for Age, Gender, Job Classification

## References

[B1] Lipsitch M (2005). Pandemic flu: we are not prepared. MedGenMed.

[B2] U.S. Department of Health and Human Services (2005). HHS Pandemic Influenza Plan.

[B3] Qureshi KA, Merrill JA, Gershon RR, Calero-Breckheimer A (2002). Emergency preparedness training for public health nurses: a pilot study. J Urban Health.

[B4] Shapira Y, Marganitt B, Roxiner I, Scochet T, Bar Y, Shemer J (1991). Willingness of staff to report to their hospital duties following an unconventional missile attack: a state-wide survey. Isr Med Sci.

[B5] Qureshi K, Gershon RR, Sherman MF (2005). Health care workers' ability and willingness to report to duty during catastrophic disasters. J Urban Health.

[B6] Dausey DJ, Lurie N, Diamond A Public Health Response to Urgent Case Reports. Health Aff (Millwood).

[B7] Sandman PM, Miller PM, Johnson BB, Weinstein ND (1993). Agency communication, community outrage, and perception of risk: three simulation experiments. Risk Anal.

[B8] Slovic P, Fischoff B, Lichtenstein S, Kates RW, Hohenemser C, Kasperson J (1985). Characterizing Perceived Risk. Perilous Progress: Managing the Hazards of Technology.

[B9] Barnett DJ, Balicer RD, Blodgett DW, Everly GS, Omer SB, Parker CL, Links JM (2005). Applying Risk Perception Theory to Public Health Workforce Preparedness Training. J Public Health Manag Pract.

[B10] Perry RW (2003). Incident management systems in disaster management. Disaster Prevention and Management.

[B11] U.S. Bureau of the Census Population Estimates Program (PEP) Updated annually.

[B12] National Association of County and City Health Officials (2001). Local Public Health Agency Infrastructure: A Chartbook.

[B13] (Anonymous) (2004). World is ill-prepared for "inevitable" flu pandemic. Bull World Health Organ.

[B14] United States Department of Homeland Security (2004). National Response Plan.

[B15] Columbia University School of Nursing Center for Health Policy (2002). Bioterrorism and Emergency Readiness: Competencies for All Public Health Workers.

[B16] Maripolsky V (2002). In disaster's aftermath, don't forget the needs of employees. Patient Care Manag.

